# GRP Receptor Regulates Depression Behavior *via* Interaction With 5-HT2a Receptor

**DOI:** 10.3389/fpsyt.2019.01020

**Published:** 2020-01-28

**Authors:** Dan Xiang, Huiling Wang, Siqi Sun, Lihua Yao, Ruiting Li, Xiaofen Zong, Gaohua Wang, Zhongchun Liu

**Affiliations:** Department of Psychiatry, Renmin Hospital of Wuhan University, Wuhan, China

**Keywords:** 5-HT2a receptor, gastrin-releasing peptide receptor, chronic unpredictable mild stress, interaction, depression

## Abstract

**Objective:**

Accumulating evidences indicate that gastrin-releasing peptide receptor (GRPR) may contribute to the pathophysiology of depression. However, the mechanism of the involvement of GRPR in the progression of depression remains unclear. Here, we showed the extent to which stress and antidepressant treatment impact GRPR expression, and explored the interactions between 5-HT2a receptor (5-HT2aR) and GRPR at the cellular level.

**Methods:**

The rat depression models were created with chronic unpredictable mild stress (CUMS). Then, these rats were treated with ﬂuoxetine for 4 weeks after CUMS. We measured body weight and performed behavioral tests to determine the effects of stress and fluoxetine on depressive-like behaviors. Real-time PCR and western blotting were used to measure the mRNA and protein expression levels of GRPR in the hypothalamus. Then, Flag-tagged protein (pcmv-Flag-5HT2aR) and Myc-tagged protein (pcmv-Myc-GRPR) expression vectors were constructed, identified, and transfected into human embryo kidney 293 (HEK293) cells. The interaction between 5-HT2aR and GRPR was detected by coimmunoprecipitation and double-label immunofluorescence.

**Results:**

The rats subjected to 4 weeks of CUMS showed depressive-like behaviors, including decreased body weight, sucrose preference, and distance traveled, rearing frequency and velocity in the open field test and increased immobility time in the forced swimming test. Fluoxetine treatment reversed CUMS-induced depressive-like behavior. The mRNA and protein expression of GRPR in the hypothalamus was significantly increased after 4 weeks CUMS exposure, and treatment with fluoxetine reversed these changes. Coimmunoprecipitation showed that 5-HT2aR and GRPR combine with each other *in vitro*. Immunofluorescence revealed that the 5-HT2aR and GRPR were colocalization in both the cell membrane and cytoplasm.

**Conclusion:**

Our study enhances the understanding of the involvement of GRPR in depression. This study also provides *in vitro* experimental evidence of the interaction between 5-HT2aR and GRPR, which may play an important role in the pathogenesis of depression.

## Introduction

Depression is a common and complex mental disorder. It is associated with enormous adverse effects in humans and high costs to society and healthcare systems (1, 2), and the pathogenesis of depression is unclear. Over the past 30 years, 5-HT has been a major target of antidepressant drugs, such as 5-HT reuptake inhibitors (SSRIs), the antidepressant effects of which may involve many types of 5-HT receptors (3). Dysfunction of the serotonergic system is closely related to the pathogenesis of depression, and several genetic studies have focused on genes encoding 5-HT2a receptor (5-HT2aR) (4, 5). The 5-HT2aR is a subtype of the 5-HT2 receptor and belongs to the seven transmembrane-spanning receptor family, which is coupled *via* G q/11 to the inositol triphosphate (IP3)/protein kinase C (PKC)/calcium pathway. 5-HT2aR is highly expressed in several brain regions that are mainly involved in the regulation of emotions, such as the hippocampus, the amygdala, the thalamus, and several cortical areas (6). In preclinical studies, 5-HT2aR mRNA and protein expression were shown to be significantly upregulated in the frontal cortex of stressed rats (7). An increasing number of studies have found the antidepressant-like effects of 5-HT2aR selective antagonists in rodents (8–10). Moreover, increased 5-HT2aR density has been confirmed in depressed patients (11). Postmortem studies have also shown increased 5-HT2aR in unmedicated depressed patients (12). Together, these studies highlight the important roles of 5-HT2aR in the pathology of depression.

Gastrin-releasing peptide receptor (GRPR) belongs to the G-protein coupled receptor (GPCR) superfamily and plays a role in several aspects of emotional responses (13). GRPR is a type of bombesin receptor in humans, mice, and rats that consists of 384 amino acids and was cloned from 3T3 cells. GRPR is directly coupled to the Gq type of G protein and is primarily associated with an increased cellular (Ca2+) and activation of the phospholipase C (PLC)/PKC and extracellular signal-regulated protein kinase (ERK)/mitogen-activated protein kinase (MAPK) pathways (14). Gastrin-releasing peptide (GRP) acts by binding to the GRP receptor, and consistent evidence has proposed that GRP might act as a stress mediator. Merali et al. found that chronic restraint exposure is associated with increased levels of GRP in the anterior pituitary (15). Rats given a systemic injection of corticosterone show enhanced release of GRP in the amygdala and medial prefrontal cortex in response to an acute stressor (16). Furthermore, several studies have shown that the dysfunction of the hypothalamic pituitary adrenal (HPA) axis is mainly involved in the course and progression of depression (17). Considerable evidence suggests that the expression of GRPR in stress-related brain areas including the hypothalamus, hippocampus, and amygdala is involved in the regulation of the HPA axis (18, 19). These data demonstrate the critical role of the GRP/GRPR system in the modulation of depressive-like behavior.

Previous studies have shown that GRP binds preferentially to GRPR, which increases 5-HT neuronal activity in the paraventricular nucleus (PVN) (20). In our previous study, we observed that GRPR mRNA and protein levels are markedly increased in the hypothalamus of CUMS-exposed mice and that treatment with ﬂuoxetine reverses these changes (21). SSRIs are effective in the treatment of depression. There are different families and subtypes of 5-HT receptors, and 5-HT2aR may be involved in the antidepressant effects of SSRIs (22). The administration of ﬂuoxetine and a reduction in either 5-HT2AR or GRPR is associated with a reduction in depression behavior. However, little is known about the molecular mechanisms of the interaction between these two important neurotransmitter systems. In this study, we used the chronic unpredictable mild stress (CUMS) to establish a depressive-like phenotype, and treatment with the antidepressant fluoxetine. We performed the behavioral tests to detect the effects of stress and fluoxetine on anhedonia and activity. We measured the mRNA and protein expression levels of GRPR in the hypothalamus. Human embryo kidney 293 (HEK 293) cells have become the mammalian cell line of choice for the production of recombinant proteins because they are easy to culture and exhibit high transfection efficiency. Transient expression in HEK293 cells provides a way of rapidly assessing the protein function. Therefore, in this study, Flag-tagged protein (pcmv-Flag-5HT2aR) and Myc-tagged protein (pcmv-Myc-GRPR) protein expression vectors were constructed, identified, and transfected into HEK293 cells. Coimmunoprecipitation and double immunofluorescence were used to explore the interaction between 5-HT2aR and GRPR at the cellular level.

## Material and Methods

### Animals

Male Sprague Dawley (SD) rats, weighing 180 g to 200 g, were obtained from the Company of Experimental Animals of Hunan slilaike jingda. The rats were housed in cages and maintained in a standard animal room (12 h/12 h light/dark cycle; 22 ± 2°C; food and water *ad libitum*). Before the CUMS procedure, the rats were acclimated to the environment for 1 week. All procedures were carried out in accordance with the guidelines of the P.R. China legislation on the ethical care and use of laboratory animals, and the Institutional Animals Care Committee of Renmin Hospital of Wuhan University approved the experimental protocols.

### Experimental Groups and Drug Treatment

**Figure 1:**
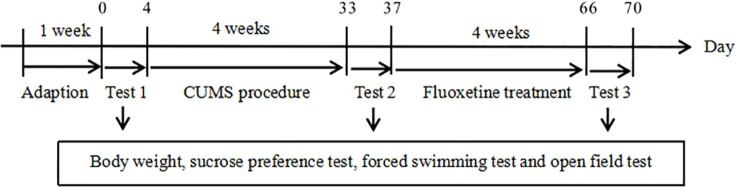
Experimental procedures.

The rats were randomly divided into three groups (n = 10/group): the chronic unpredictable mild stress (CUMS) + normal saline group (CUMS group); the CUMS + fluoxetine group (fluoxetine group); and the control group. At the end of the CUMS procedure, normal saline was administered daily to the CUMS group rats for 4 weeks by intraperitoneal injection. Fluoxetine (Aladdin; F131623) was diluted in normal saline and intraperitoneally administered to the fluoxetine group rats. A previous work showed that a 4-week course of fluoxetine at a dose of 10 mg/kg produces antidepressant effects. The experimental design is shown in **Figure 1**.

### CUMS Procedure

After 1 week of the acclimatization period, the depression model was established by CUMS as described previously with slight modification (23). The rats in the CUMS group and fluoxetine group were subjected to seven different stressors for 4 weeks. They were exposed to food deprivation for 24 h; water deprivation for 24 h; 45° cage tilt for 24 h; swimming in 4°C ice water for 5 min; tail clamping for 3 min; damp sawdust for 24 h; lights on overnight. Each animal received one of these stressors per day and the same stressor was not presented consecutively over 2 days.

### Body Weight and Behavioral Tests

We measured the weights of the rats before and after the CUMS procedure, and the rats were again weighed after the fluoxetine treatment. The sucrose preference test, forced swimming test, and open field test were conducted before the CUMS procedure began, at the end of the 4-week CUMS period and after fluoxetine was administered. The sucrose preference test was performed as described previously with minor modifications to quantify anhedonia (24). Before the test, the rats were trained to consume sucrose solution (1%). After adaptation, the rats were deprived of water for 24 h. Subsequently, each rat was given two bottles, one containing 1% sucrose solution and the other containing tap water, for 24 h (the positions of the bottles were changed after 12 h). Sucrose preference was calculated by the following formula: sucrose preference (%) = (sucrose intake/total fluid intake) × 100%. In the forced swimming test, the rats were placed individually in a cylindrical tank that they were unable to exit. The immobility time, the length of time during which the rats remained still without struggling or used only minor movements to keep themselves afloat, was recorded. Each rat was forced to swim for 6 min, and the total time spent immobile during the final 4 min was recorded. The open field test was used to detect the spontaneous activity of the rats. The apparatus consisted of a square 100 × 100 cm area with 35-cm high walls. A rat was placed in the center of the rectangular cage and observed using a video tracking system for 5 min (Ethovision XT 11.5). The parameters that were assessed were the distance traveled, the speed and the frequency of rearing.

### Sample Collection

The samples were collected after the final behavioral tests. The hypothalamus was immediately isolated after the rats were sacrificed under deep anesthesia. The samples were stored at −80°C until use.

### Plasmid Construction

5-HT2aR and GRPR cDNA sequences were synthesized by the Wuhan Tianyi Huiyuan Company. The 5-HT2aR and GRPR genes were amplified by polymerase chain reaction (PCR), and the following primer sequences were used: 5-HT2aR (forward: 5’-CCCAAGCTTATGGAAATTCTCTGTG-3’; reverse: 5’-CCGGAATTCTCACACACAGCTAACC-3’) and GRPR (forward: 5’-CAGCTCGAGATGGCTCCAAATAAT-3’; reverse: 5’-CCGGAATTCCTAGACATACCCCT-3’). The PCR conditions were as follows: 1 cycle of 95°C for 5 min and then 38 cycles of 95°C for 15 s, 56°C for 15 s, and 72°C for 15 s. The PCR products were analyzed by 1% agarose gel electrophoresis and purified with a DNA purification kit (OMEGA; D2500-01). Then, 5-HT2aR cDNA was subcloned into the pcmv-flag vector at the EcoR I and Hind III sites, and the GRPR cDNA was subcloned into the pcmv-myc vector at the EcoR I and Xho I sites. The plasmids were transformed into DH5α by the heat shock method and extracted with a plasmid mini kit (OMEGA; D6943-01). Finally, the pcmv-Flag-5HT2aR and pcmv-Myc-GRPR plasmids were identified by restriction enzyme digestion.

### Cell Culture and Transfection

HEK293 cells were obtained from the microbiology laboratory of the School of Basic Medicine, Wuhan University. The cells were cultured in DMEM (Gibco, 21885108) supplemented with 10% FBS and gentamicin and incubated at 37°C in 5% CO_2_. The cells were seeded in 10-cm culture dishes 24 h before transfection. The pcmv-Flag-5HT2aR and pcmv-Myc-GRPR plasmids were cotransfected with Lipofectamine 2000 reagent (Thermo,11668019) according to the manufacturer’s instructions. Single transfection of the pcmv-Flag-5HT2aR or pcmv-Myc-GRPR plasmid was performed in the control groups.

### RNA Extraction and RT-PCR

Total RNA was extracted from the hypothalamus using Trizol reagent (Ambion, 15596-026) and purified according to the manufacturer’s instructions. The RNA concentration was determined by spectrophotometer at A260/A280 nm. Reverse transcription was performed with mRNA (3 µg) using a first-strand synthesis kit (Thermo, K1691) for cDNA synthesis. cDNAs were subsequently amplified by PCR with the following specific primers: GAPDH (forward: 5’-TGTGAAGCTCATTTCCTGGTATG-3’, reverse: 5’-AGGGCCTCTCTCTTGCTCTC-3’) and GRPR (forward: 5’-GCAGGATTGGCTGCAAACTG-3’, reverse: 5’-ATTGGCCTGACGATGGCTTT-3’). PCR was carried out as follows: 1 cycle of 95°C for 5 min followed by 39 cycles of 95°C for 10 s, 60°C for 30 s, and 72°C for 30 s, and finally 72°C for 10 min. The expression of GRPR mRNA was analyzed by 2^-ΔΔCT^ method, and was normalized to GAPDH as a reference gene. Each experiment had more than three independent replicates.

### Protein Extraction and Western Blotting

Western blotting was performed to test the expression of GRPR in the hypothalamus of rats and the cultured HEK 293 cells. Total protein from each group was extracted in 1 ml of RIPA buffer (Beyotime, P0013B) with 1% PMSF and protease inhibitor cocktail. The hypothalamus tissues were homogenized in ice cold RIPA lysis buffer. The HEK293 cells were washed with PBS 48 h after transfection and lysed with ice cold RIPA lysis buffer. Tissues and cells were centrifuged for 15 min at 12,000 rpm at 4°C, and the supernatants were collected and stored at −80°C. The concentration of proteins was determined by the BCA assay (BCA Protein Assay Kit, Thermo,23228). Then, 12% polyacrylamide gel electrophoresis was used to separate the protein samples, and then the proteins were transferred onto PVDF membranes (Merck millipore, ISEQ00010). The PVDF membranes were blocked in 5% non-fat dry milk at room temperature for 1 h. Then, the membranes were incubated with anti-GRPR antibody (1:200) (Santa Cruz, sc-32904), anti-Flag antibody (1:2,000) (Abbkine; A02010), and/or anti-Myc antibody (1:2,000) (Abbkine; A02061) at 4°C overnight. The following day the membranes were washed with TBST for three times. Then the membranes were incubated with secondary antibodies (1:5,000) at room temperature for 2 h. The immunoreactions were visualized with a chemiluminescence kit (Beyotime, P0018AM). GAPDH was used as a loading control to analyze the relative protein expression of GRPR. The intensity of the protein bands was calculated by ImageJ software. Each experiment was performed more than three times.

### Coimmunoprecipitation Analysis

The protein samples were preincubated with anti-Flag antibody (1:200) at 4°C overnight. Then, 50% agarose beads were washed with PBS, incubated at 4°C for 2 h, centrifuged to remove the nonspecific binding protein, and added to the samples. Rabbit IgG was used as a negative control. The antigen-antibody complexes proteins were separated using 10% polyacrylamide gel electrophoresis and transferred onto PVDF membranes. The membranes were blocked with 5% non-fat dry milk for 1 h, and were incubated with anti-Myc antibody (1:2,000) at 4°C overnight. Similarly, the protein samples were preincubated with anti-Myc antibody (1:200), and the antigen-antibody complex proteins were detected by anti-Flag antibody (1:2,000). Finally, the immunoreactions were performed with a chemiluminescence kit after incubation with secondary antibodies (1:5,000).

### Double-Label Immunofluorescence and Confocal Microscopy

Double-label immunofluorescence was used to analyze 5-HT2aR and GRPR distribution in HEK293 cells. After 48 h of the transfection, HEK293 cells were washed with PBS, fixed with 4% paraformaldehyde, and permeabilized with 0.3% Triton X-100. The cells were blocked with 5% non-fat dry milk for 1 h. Then, the cells were incubated in a mixture of mouse anti-Flag monoclonal antibody (1:200) and rabbit anti-Myc polyclonal antibody (1:200) overnight at 4°C. The second day, the cells were washed with PBS several times and incubated with DyLight488-conjugated goat anti-mouse IgG (Abbkine; A23210) and DyLight594-conjugated goat anti-rabbit IgG (Abbkine; A23420) in the dark at room temperature for 1 h. Nuclei were stained with DAPI for 5 min. After staining, the fluorescence signals were examined with a laser-scanning confocal microscope.

### Statistical Analysis

Statistical analysis was performed using SPSS 20.0 software, and all data were reported as the means ± standard error of mean (SEM). The data were analyzed by one-way ANOVA test followed by LSD *post hoc* test. Statistical significance was set at *P* < 0.05.

## Results

### Body Weight and Behavioral Tests

**Figure 2:**
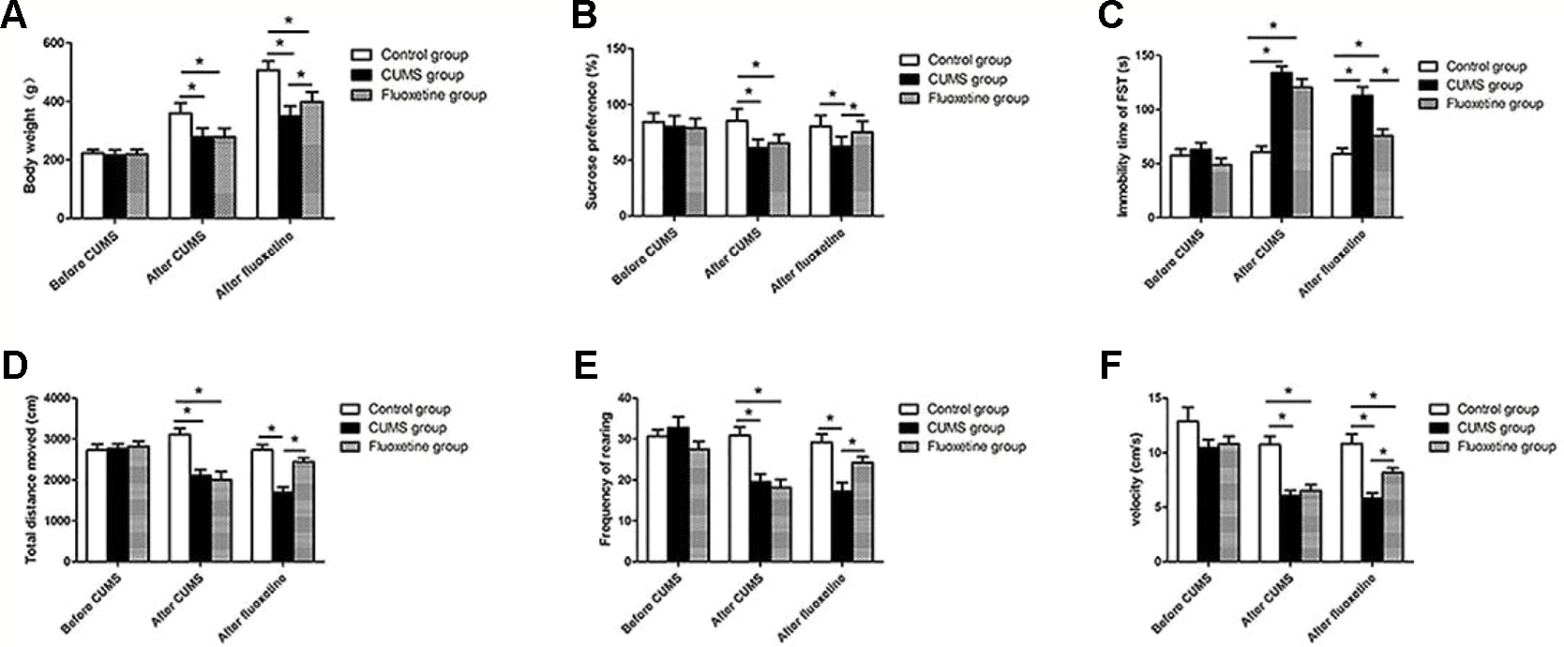
Body weight **(A)**, sucrose preference **(B)**, forced swimming test **(C)**, total distance moved **(D)**, rearing frequency **(E)**, and velocity **(F)**. *P < 0.05.

As shown in **Figure 2**, before the CUMS procedure, no significant difference was observed among the three groups, but the CUMS group and the fluoxetine group were significantly different from the control group following 4 weeks of CUMS. The CUMS group and the fluoxetine group rats showed a lower index of body weight (F = 43.48, *P* < 0.05) and sucrose preference (F = 102.94, *P* < 0.05), decreased distance traveled (F = 12.93, *P* < 0.05), rearing frequency (F = 12.14, *P* < 0.05) and velocity (F = 17.87, *P* < 0.05) in the open field test and an increased immobility time in the forced swimming test (F = 35.75, *P* < 0.05). Compared with the CUMS group, the fluoxetine groups exhibited significantly increased the body weight (F = 120.14, *P* < 0.05), sucrose preference (F = 68.11, *P* < 0.05), and the distance traveled (F = 20.34, *P* < 0.05), rearing frequency (F = 10.48, *P* < 0.05), and velocity (F = 16.13, *P* < 0.05) in the open field test and decreased immobility time in the forced swimming test (F = 16.70, *P* < 0.05).

### GRPR mRNA Expression

**Figure 3:**
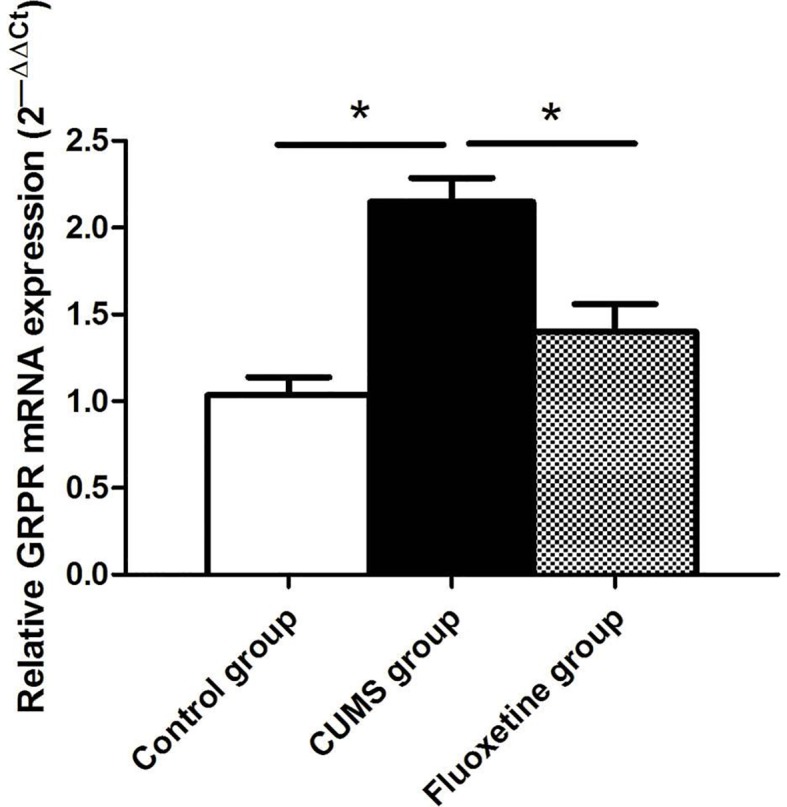
GRPR mRNA expression in the hypothalamus between groups. *P < 0.05.

As shown in **Figure 3**, significant differences in the mRNA expression of GRPR were observed in the hypothalamus of rats between groups. Compared with that in rats from the control group, the mRNA expression of GRPR was significantly increased in rats from the CUMS group, and fluoxetine treatment significantly downregulated the mRNA expression of GRPR in rats from the fluoxetine group (F = 17.32, *P* < 0.05).

### GRPR Protein Expression

**Figure 4:**
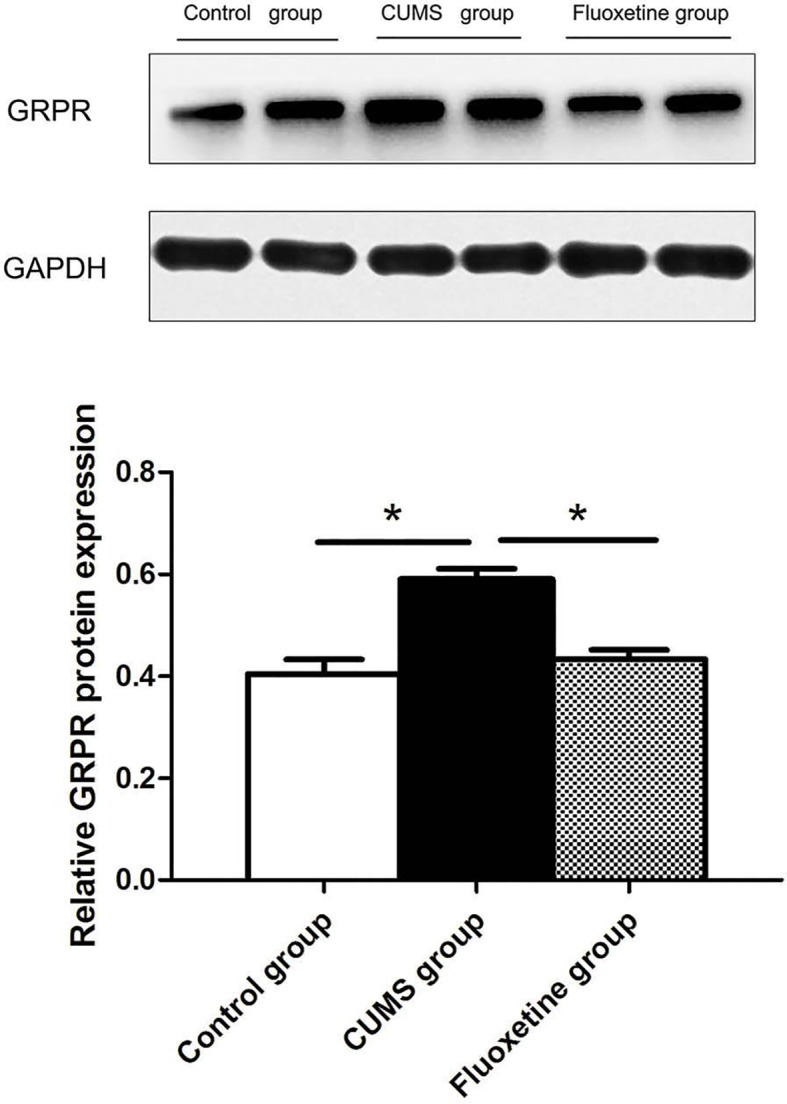
GRPR protein expression in the hypothalamus between groups. *P < 0.05.

**Figure 4** shows the analysis of GRPR protein using western blotting in hypothalamic tissue. The GRPR protein level in the CUMS group was significantly increased compared with that in the control group and the fluoxetine group (F = 19.38, *P* < 0.05).

### 5-HT2aR Is a GRPR-Interacting Protein In *Vitro*

**Figure 5:**
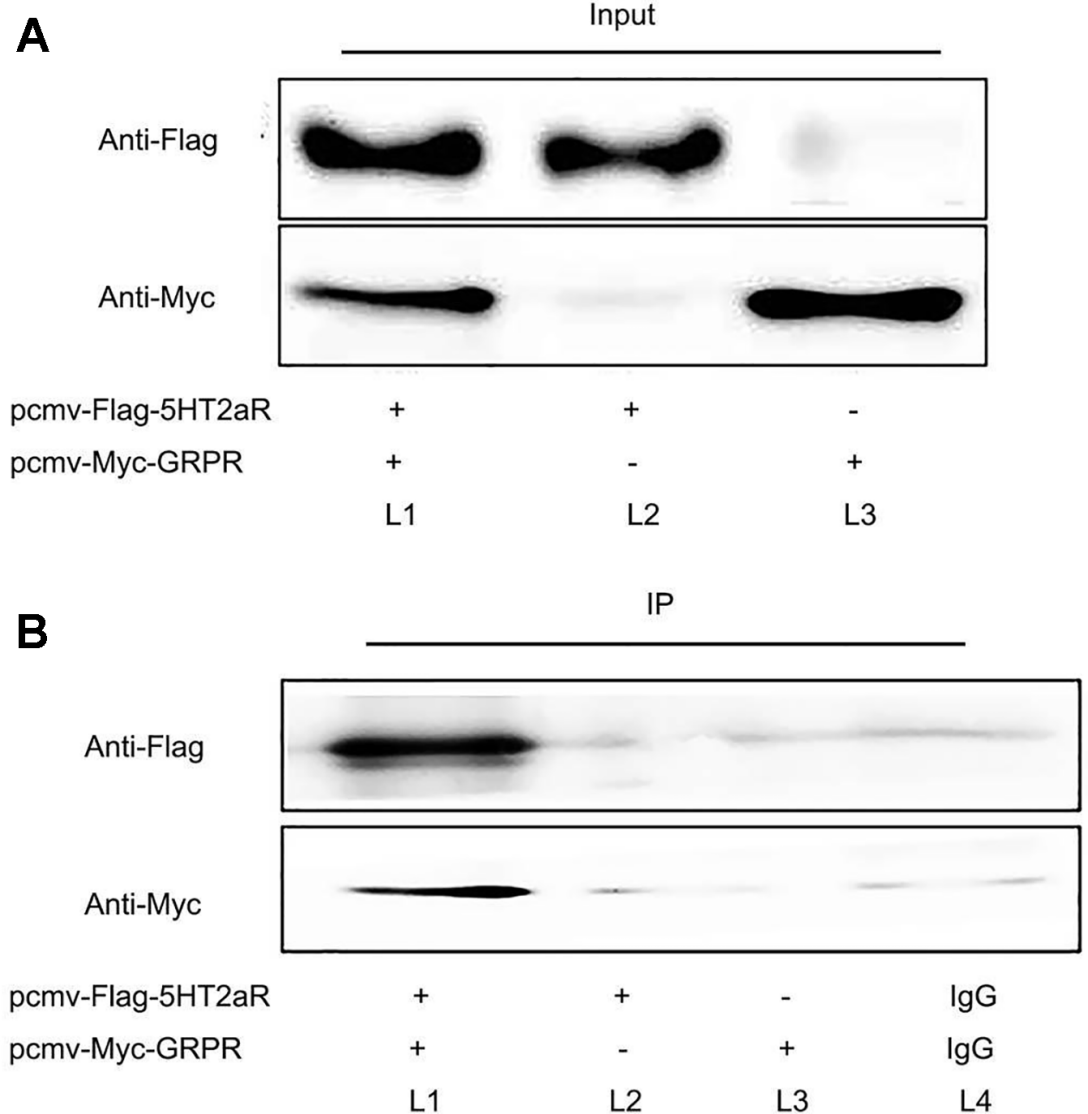
Coimmunoprecipitation of 5-HT2aR and GRPR. Input **(A)** and IP **(B)**. The pcmv-Flag-5HT2aR and pcmv-Myc-GRPR plasmids were cotransfected into HEK293 cells (L1). The pcmv-Flag-5HT2aR plasmid was singly transfected into HEK293 cells (L2). The pcmv-Myc-GRPR plasmid was singly transfected into HEK293 cells (L3). IgG served as the negative control (L4).

To investigate whether 5-HT2aR is functionally associated with GRPR, coimmunoprecipitation was performed *in vitro*. The pcmv-Flag-5HT2aR and pcmv-Myc-GRPR plasmids were constructed, identified, and cotransfected or singly transfected into HEK293 cells. First, protein from the cotransfected cells was immunoprecipitated with anti-Flag antibody and analyzed by western blotting with anti-Myc antibody. The results showed that a GRPR band was detected in the immunoprecipitate of the anti-Flag antibody. Then, anti-Myc antibody was used for immunoprecipitation, and anti-Flag antibody was used for western blotting. A 5-HT2aR band was detected in the immunoprecipitate of the anti-Myc antibody. However, neither a 5-HT2aR band nor a GRPR band were detected in singly transfected cells immunoprecipitated with antibodies for both proteins. The protein bands were also not present in the immunoprecipitate of rabbit IgG (**Figures 5A, B**).

### Colocalization of 5-HT2aR and GRPR

**Figure 6:**
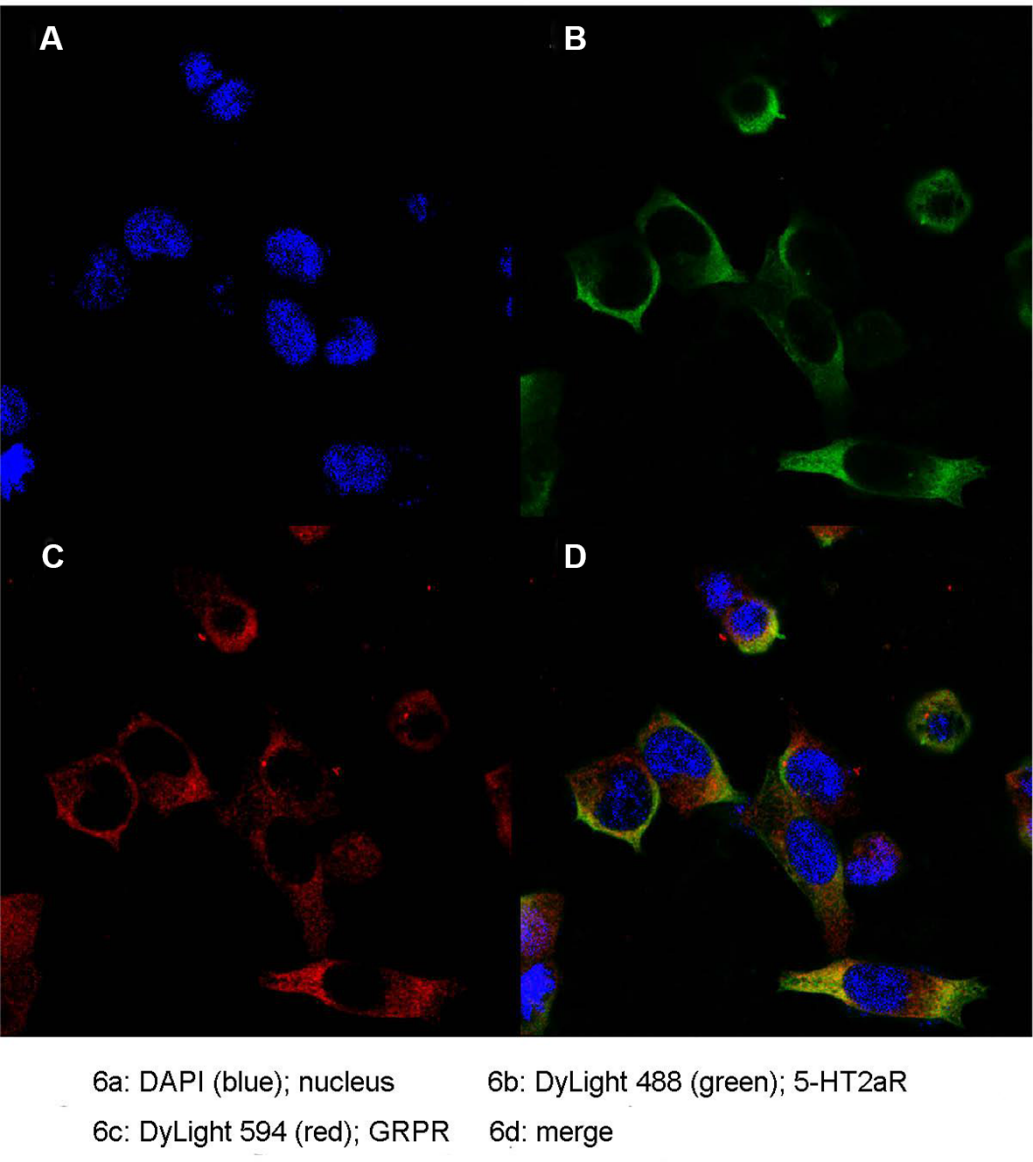
Colocalization of 5-HT2aR and GRPR. Nucleus **(A)**; 5-HT2aR **(B)**; GRPR **(C)**; merge **(D)**.

To further determine the expression pattern of 5-HT2aR and GRPR, we used the anti-Flag antibody and anti-Myc antibody specifically bound to HEK293 cells cotransfected with pcmv-Flag-5-HT2aR and pcmv-Myc-GRPR plasmids. Double immunofluorescence staining for 5-HT2aR and GRPR revealed that the expression of the two receptors overlapped in HEK293 cells. As revealed by confocal microscopy and shown in **Figure 6**, 5-HT2aR and GRPR were colocalized in the cytoplasm and membrane of HEK293 cells, as revealed by confocal microscopy.

## Discussion

In this study, 4 weeks of CUMS was used to establish a classical model of depression in rats. The results indicated that fluoxetine treatment had an antidepressant-like effect in a CUMS model of depression in rats. Fluoxetine treatment decreased the levels of GRPR mRNA and protein in the hippocampus of stressed rats. Our coimmunoprecipitation results showed that 5-HT2aR and GRPR combine with each other *in vitro*. Additionally, immunofluorescence results revealed that the 5-HT2aR and GRPR were colocalized in both the cell membrane and cytoplasm. Our findings provide additional research ideas to explore the pathogenesis of targeting GRPR signaling in depression. Our data also supports that a functional interaction between GRPR and 5-HT2aR, were supported at the cellular level.

CUMS is regarded as a valid animal model of depression-like behavior (25). In this study, a classical model of depression was successfully built by 4 weeks of CUMS. Anhedonia is one of the major symptoms of depression, and is manifested as a reduction in interest or pleasure in daily activities. Additionally, CUMS causes many other symptoms of depression, such as decreases in food/water intake, exploratory behaviors, and locomotor activity, and helplessness (26). A reduction in sucrose preference in stressed rats reflects anhedonia, and an increase in immobility time in the forced swimming test indicates a lower desire to escape, which may be similar to the helplessness symptom in depression (27). The lower body weight observed in the stressed rats suggests that CUMS induced a decrease in food/water intake. In the open field test, CUMS significantly reduced the distance moved, velocity, and rearing frequency of rats. These results indicated that stressed rats exhibited less activity and fewer exploratory behaviors in new environments (28). Fluoxetine treatment led to a significant improvement in body weight, sucrose preference, and distance traveled, rearing frequency, and velocity in the open field test, and a decrease in immobility time of the forced swimming test. These findings are in agreement with previous studies and suggest that fluoxetine has antidepressant-like effects.

In this experiment, we showed that the mRNA and protein expression of GRPR in the hypothalamus were significantly upregulated in CUMS rats and that the expression of GRPR was downregulated after fluoxetine treatment. GRP binds preferentially to GRPR and stimulates the release of adrenocorticotropic hormone (ACTH) and corticosterone, and increasing the activity of the HPA axis, evokes behaviors associated with stress (29). GRP and GRPR are present in several brain regions implicated in the stress response, including the amygdala, hippocampus, hypothalamus, and bed nucleus of the stria terminalis, as well as caudal brainstem structures such as the nucleus tractus solitarii, parabrachial nucleus, and locus coeruleus (30). In addition, studies have shown that the locomotor activity and non-aggressive social behaviors are increased in the GRPR-deficient mice (31). In this study, we further confirmed that the mRNA and protein expression of GRPR was increased in CUMS rats. The results also showed that chronic ﬂuoxetine treatment restored the stress-induced increase in GRPR expression. However, the mechanisms by which ﬂuoxetine treatment restores GRPR expression remain unclear.

Fluoxetine is an SSRI and exerts its pharmacological effects through the manipulation of the 5-HT system (32). Fluoxetine selectively block 5-HT transporters, thereby increasing extracellular concentrations of 5-HT at the postsynaptic 5-HT receptors (33). In addition, fluoxetine treatment induces a complex array of neuropharmacological changes, such as a reduction in the density of 5-HT2aR (34). A Japanese cohort study suggested that 5-HT2aR may play an important role in the pathophysiology of the therapeutic response to SSRIs (35). In rats, chronic treatment with citalopram decreases the 5-HT2aR density in the brain cortex (36). Additionally, several studies have found that the CUMS-induced increase in 5-HT2aR expression can be decreased by fluoxetine administration (37, 38). Moreover, Qesseveur et al. explored the genetic variants of the 5-HT2aR gene that affect the therapeutic outcome of antidepressants. The results suggested that the genetic inactivation of the 5-HT 2A receptor may affect the antidepressant effects of SSRIs (39). These findings suggest that 5-HT2aR may be involved in the mechanism of action of antidepressant effect. In our study, we also found that chronic fluoxetine treatment decreased GRPR expression in the hypothalamus of rats. To explore the mechanism underlying the regulation of this response, we used HEK293 cells to explore the interactions between 5-HT2aR and GRPR. We used coimmunoprecipitation to identify the protein-protein interaction between 5-HT2aR and GRPR and used double immunofluorescence staining to study the colocalization of 5-HT2aR and GRPR *in vitro*. By performing coimmunoprecipitation and double immunofluorescence staining, we confirmed the interaction between 5-HT2aR and GRPR *in vitro*, which may provide new ideas for the treatment of depression.

One limitation is that our study was only performed *in vitro*, so it may not fully explain the possible mechanism of the interaction between these two receptors in the pathogenesis of depression. Therefore, it is necessary to confirm this conclusion in patients or animal models of depression. Another limitation is that we only explored the impacts of stress and fluoxetine treatment on GRPR expression. GRPR plays a physiological role by activating the PLC/PKC pathway, has a similar signaling pathway to 5-HT2aR. The molecular and cellular basis for GRPR-5-HT2aR cross-signaling may be an interesting research direction in this field.

In general, this study confirmed a change in GRPR expression in the rat hypothalamus after stress. Additionally, for the first time, this study provides experimental evidence of the interaction between 5-HT2aR and GRPR *in vitro*. These results highlight the involvement of GRPR in depression, and provide a novel biological target for the treatment of depression.

## Data Availability Statement

All datasets generated for this study are included in the article/supplementary material.

## Ethics Statement

The animal study was reviewed and approved by The P.R. China legislation on the ethical care and use of laboratory animals, and the Institutional Animals Care Committee of Renmin Hospital of Wuhan University.

## Author Contributions

ZL and GW designed and supervised the study. DX carried out the experimental procedures and analyzed the date. ZL and DX interpreted results of experiments and drafted the manuscript. ZL revised the manuscript. All authors provided feedback on manuscript.

## Funding

This work was supported by the National Key R&D Program of China (2018YFC1314600) and the National Natural Science Foundation of China (81771472, 81271496, and 30971040).

## Conflict of Interest

The authors declare that the research was conducted in the absence of any commercial or financial relationships that could be construed as a potential conflict of interest.
